# Consumer Acceptance of Cultured Meat in Romania Highlighting Sustainable Perspectives for Both Human and Pet Consumption

**DOI:** 10.3390/ani15192867

**Published:** 2025-09-30

**Authors:** Silvia-Ioana Petrescu, Mădălina Matei, Cristina-Gabriela Radu-Rusu, Andrei Ciobanu, Dragoș Mihai Lăpușneanu, Ioan Mircea Pop

**Affiliations:** “Ion Ionescu de la Brad” Iasi University of Life Sciences, 3 Mihail Sadoveanu Alley, 700489 Iasi, Romania; silvia.petrescu@iuls.ro (S.-I.P.); cristina.radurusu@iuls.ro (C.-G.R.-R.); andrei.ciobanu@iuls.ro (A.C.); dragos.lapusneanu@iuls.ro (D.M.L.); mircea.pop@iuls.ro (I.M.P.)

**Keywords:** cultured meat, Romania, food, feed, sustainability

## Abstract

**Simple Summary:**

As demand for sustainable food alternatives grows, cultured meat is emerging as an innovative option that can reduce pressure on natural resources. In this study, we analyzed the attitude of consumers in northern Romania towards the consumption of cultured meat, both for their own diet and for pet food. The results revealed a high level of acceptance, with over 70% of respondents in favour of introducing cultured meat to the market under regulation, and over 30% indicating that they would feed it to their pets. Perceptions were influenced by demographic differences, such as gender, age, and place of residence: women emphasised ethical and environmental aspects, while men emphasised safety and scientific validation. These results suggest that promoting cultured meat in Romania could benefit from communication strategies tailored to different audience segments.

**Abstract:**

Cultured meat is an emerging innovation with the potential to contribute to sustainable animal production by reducing the environmental and ethical challenges associated with conventional animal farming. In Romania, this new source of protein is viewed with both interest and scepticism, reflecting broader consumer uncertainties about food innovations. Market data indicates that Romania faces a significant deficit in animal protein supply (exceeding 65% for pork, as well as notable deficits in fish and beef), while also recording one of the largest overall trade deficits in pet food. In this context, the present study explored the openness of respondents in northeastern Romania to the consumption of cultivated meat, taking into account both personal diet and pet nutrition. The results showed that 33.2% of participants were fully willing to feed their pets cultured meat products, and 70% supported its introduction to the market, provided that regulatory frameworks ensure product safety and quality. Although Romania does not currently have the legal and technological infrastructure necessary to integrate cultured meat into the food system, these findings highlight the potential role of consumer acceptance in shaping future nutritional strategies aimed at sustainable, high-quality protein alternatives for humans and pets alike.

## 1. Introduction

Cultured meat, also known as cell-based meat, is an innovative form of animal protein obtained by cultivating animal cells in a controlled environment. It has the potential to include products such as seafood and organs [1]. This emerging technology has the potential to provide products that are comparable to conventional animal-based foods in terms of nutrition and sensory properties, while helping to reduce pressure on natural resources and address ethical concerns about animal farming [2,3].

According to the FAO and WHO, cultured meat could be a viable solution to future food security and environmental challenges, given the substantial forecast growth in global meat demand in the coming decades [2,3]. However, recent studies emphasize that the technology’s potential hinges on overcoming significant technological challenges. These include obtaining stable cell lines, developing serum-free or low-serum culture media, optimizing cell proliferation and differentiation processes, and designing cost-effective bioreactors and scaffolds [4,5]. While current research has made progress in using edible scaffolds, 3D printing and nanofibres to reproduce the texture and structure of conventional meat, these technologies are still far from being produced on an industrial scale.

Recently, regulatory measures have been taken in several countries to authorize the use of cultured meat products for human and animal consumption. In the international context, in 2020 Singapore became the first country to approve the use of cultured meat for human consumption by authorizing a hybrid minced chicken product containing vegetable protein and 3% cultured chicken [5,6]. In 2023, the United States approved the use of cultivated chicken meat for human consumption, but its marketing was initially restricted to tasting sessions organised in selected restaurants. In 2024, Israel granted approvals for the human consumption of cultivated beef steak. Also in 2024, Singapore and Hong Kong authorised the consumption of cultivated quail meat. In the United Kingdom, in the same year, the use of cultivated chicken meat in pet products, in the form of dog and cat treats, was approved [7,8,9].

In terms of national dynamics, according to one of the most recent surveys conducted in 15 European countries, including Romania, 49% of respondents in Romania would agree to the sale of cultivated meat, provided that it passes the assessment carried out by food safety regulatory authorities. Also, 53% of respondents say that meat consumption in Romania is high, but in the next two years, 54% of them plan to cut back on meat [10].

In Romania, meat consumption patterns vary depending on the region and local traditions. In particular, in the north-east, corresponding to the historical region of Moldova, food preferences are predominantly oriented towards lean meat, especially poultry and fish [11,12] due to cultural influences and local culinary customs. However, pork, veal, mutton and game continue to be consumed frequently, being integrated into various traditional dishes [11,13]. The consumption of lamb and mutton is lower and occurs mainly during the Easter holidays, when traditional dishes include these types of meat. This trend indicates occasional consumption rather than a constant presence in the daily diet of the population in this region [14]. Some authors have noted a tendency among residents in the aforementioned area to consume lean meat, especially poultry and fish. This trend appears to be influenced by economic factors, such as the lower price of poultry compared to pork or beef, as well as health reasons and cultural perceptions related to foods that are more easily digestible or less fatty [15,16]. For instance, Marin et al.’s [17] study indicates that chicken is favoured due to its affordability and high digestibility. Recent surveys suggest that health is one of the main reasons for reducing consumption of red or fatty meat.

The main purpose of this study was to analyze how open respondents in north-eastern Romania were to consuming cultivated meat. Although the technology is still in its infancy, and there is currently no legislation or infrastructure in Romania to allow such products to be sold, this study anticipates consumer receptivity to emerging food innovations. Secondarily, perceptions of cultured meat as a possible future pet food option were explored, given that this is an emerging discussion in the field and that the first products containing small amounts of cultured meat have already appeared internationally. A relevant indication in this regard is the fact that, although few in number, some respondents say they already feed their animals insect-based protein, reflecting a positive attitude towards alternative and sustainable solutions in animal nutrition.

## 2. Materials and Methods

This study is quantitative, descriptive, and cross-sectional, aiming to investigate Romanian consumers’ perceptions of cultured meat, both in the context of human consumption and use in pet food.

An online questionnaire, developed using the Google Forms platform, a service provided by Google LLC (Mountain View, California, USA), was used to collect data. Its structure and content are presented in descriptive form in Table 1. The questionnaire was written in Romanian and was addressed exclusively to people of Romanian origin, with access distributed through social networks and other digital channels, with the aim of attracting respondents from different age, gender, education, and environmental backgrounds.

The survey questions were adapted based on examples and approaches found in international studies on the acceptance of cultivated meat. The survey targeted aspects such as attitudes and consumption intentions, acceptability in relation to conventional meat, and perceptions of safety and health [18,19,20,21]. Only respondents owning at least one pet were included in the study. Additionally, items were included to capture consumer attitudes towards pet food, based on a study conducted in Italy [22], as well as towards alternative protein sources. To verify the clarity of the Romanian version of the questionnaire, an informal pilot study was conducted with 20 participants. The research instrument consisted of 20 questions, structured into four thematic sections, as follows:

1. General, socio-demographic, and consumer characterization information—included questions about the age, gender, education level, and diet of respondents to outline a relevant demographic profile of the sample.
2. Perceptions of cultured meat (human consumption)—this section was structured to facilitate a quick understanding of the content and focused on attitudes and levels of acceptance of cultured meat in human diets.
3. Cultured meat for pets—investigated opinions on the use of cultured meat in pet food products.
4. Conclusions and personal opinions—the last section gave respondents the opportunity to openly express their opinions on the subject. The only open-ended question in the questionnaire was included here and was optional, unlike the other questions, which were mandatory and had predefined answer options (multiple choice).

The questionnaire was available online for a limited period from February to May 2025, and respondents were selected randomly through self-selection, due to the open na-ture of distribution in the digital environment. The groups of people, mostly pet owners, were selected from online sources and private groups of pet owners within veterinary practices. Participation was voluntary and anonymous, with no personal data collected. Respondents were informed about the purpose and use of the research.

To investigate differences in perception and acceptance of cultured meat, a detailed statistical analysis was performed using the ANOVA function in Microsoft Office Excel (Microsoft Corp., Redmond, WA, USA), which is considered appropriate for simple comparisons. For analyses involving multiple comparisons between age groups (described in the section Socio-Demographic Profile of Respondents: Age Influence and highlighted in detail in Supplementary Table S1), GraphPad Prism 10.1.0 software (GraphPad Software, San Diego, CA, USA) was used, which applies the same basic statistical principles but also allows post hoc tests (Tukey) to be performed.In the questionnaire presented, the questions were numbered from Q1 to Q20. Questions Q1–Q6 collected socio-demographic information (age, gender, background, education level, diet type, and household decision-maker). These were used to describe the sample and as grouping variables in the analysis, but were not suitable for statistical testing, as they were categorical descriptors. Questions Q10–Q12 referred to pet ownership and general feeding practices; these served to classify respondents but were not formulated on a scale that allowed for statistical comparisons. Question Q20 was open-ended and provided qualitative information rather than quantitative data. Therefore, only items Q7, Q8, Q9, Q13, Q14, Q15, Q16, Q17, Q18, and Q19 directly assessed perceptions and attitudes using measurable response scales, which were subjected to statistical analysis in accordance with the research hypotheses; Table 2 indicates the response scale used for each of these questions. The sample was segmented according to three main socio-demographic variables: the gender of respondents (female/male), their place of residence (urban/rural), and age groups influence. The aim was to observe whether these characteristics significantly influence opinions on the consumption of cultured meat, both for humans and for pets.

According to national demographic data [23], the median age of Romania’s population is 43.2 years, approximately 55% of population live in urban areas. The proportion of women is slightly higher than that of men (51.5%). By contrast, our sample was characterised by a much higher proportion of women (75.1%), people under 34 (approximately 80%) and people from urban areas (75.5%), over 60% of whom had a university education.

However, it should be noted that currently, only a small percentage of Romania’s population regularly uses digital tools, which inevitably influences the structure of the respondent group.

Therefore, while the results cannot be considered representative at a national level, they do provide valuable insights into the receptivity of active online consumers to cultured meat and other emerging food innovations.

For each relevant question, descriptive statistics were calculated, including the mean, standard deviation (St. Dev.), Skewness, and Kurtosis coefficients. The methodological approach was similar to those used in comparable studies conducted in other European countries [18,21,22,24]. To test the statistical significance of the differences between groups (gender and place of residence), *t*-tests were applied, with a significance threshold of *p* < 0.05.

### Ethics Statement

Ethical approval was not required for this study in accordance with institutional and national guidelines, as the research involved only an anonymous, voluntary online survey without the collection of any personally identifiable information. Informed consent was obtained from all participants through the introductory statement: “Participation in this survey is anonymous and voluntary. No personally identifiable data is collected and the responses will be used exclusively for empirical research purposes to analyze Romanians’ perceptions of cultured meat. Continuing to complete the questionnaire constitutes your informed consent to participate.”

## 3. Results

The raw results regarding perceptions and attitudes towards cultured meat for human and animal consumption are summarized in Table 2.

### 3.1. Socio-Demographic Profile of Respondents

The questionnaire was designed to include only adults aged 18 or older, corresponding to the legal age of majority in Romania. The most represented age group was 18–24, accounting for 47.2% of all respondents. The 25–34 age group was also significantly represented, accounting for 32.6% of the total sample (*N* = 233), indicating a predominance of young people among the participants.

In terms of gender distribution, analyzing the raw results in Table 2, it can be seen that women were in the majority, representing 75.1% of all respondents.

In terms of place of residence, the urban population was predominant, with a percentage of 75.5%, reflecting both easier access to digital resources (the questionnaire being online) and a possible greater openness to food innovations in urban areas.

The analysis of educational level showed that 35.2% of participants stated that high school was their highest level of education, followed by 28.8% with a bachelor’s degree and 26.6% with postgraduate studies (master’s degree). The distribution of educational levels showed a direct correlation with age, with younger people either completing their studies or just entering university.

The respondents’ diets were also investigated to assess the potential acceptance of cultured meat based on current eating behaviors. The data showed that an overwhelming majority (86.7%) identify as omnivores, while 9% adopt a flexitarian diet, consuming meat occasionally. Vegetarians (including ovo-lacto-vegetarians) and vegans accounted for a combined 4.3% of all participants, reflecting a relatively limited openness of the general population to meat-free diets.

#### 3.1.1. Socio-Demographic Profile of Respondents: Gender Influence

In Table 3 we wanted to highlight those who are more open to accepting cultured meat, comparing the genders, so a statistical analysis was performed to show the differences in perception of cultured meat both for personal consumption and the perception of animal owners regarding cultured meat in pet products. Yet, these results require careful interpretation, given the high representation of women in the sample. They suggest that the influence of gender on the acceptance of cultured meat is nuanced and may differ depending on the cultural context and methodological approach used.

#### 3.1.2. Socio-Demographic Profile of Respondents: Environment Influence

A comparative analysis of the responses between urban and rural participants shows that in general, there are no statistically significant differences between the two groups (*p* > 0.05) as shown in Table 4.

This result suggests that perceptions, knowledge levels, and attitudes towards cultured meat are relatively similar, regardless of the respondents’ place of residence. Regarding motivations to purchase and try cultured meat (Q9), both rural and urban respondents indicated that the most important aspect is the existence of scientific evidence regarding product safety, with no statistically significant differences between the two groups.

#### 3.1.3. Socio-Demographic Profile of Respondents: Age Influence

In Table 5, Tukey’s multiple comparisons test was applied to assess differences between age groups (18–24, 25–34, 35–44, 45–54, and 55+ years) regarding responses to questions Q7, Q8, Q9, Q13, Q14, Q15, Q16, Q17, Q18, and Q19. Most differences were not statistically significant, indicating relatively similar perceptions across age groups. Only the significant comparisons are summarized in Table 5, while the complete set of results is provided in Supplementary Table S1.

### 3.2. Perception of Cultured Meat for Animal Consumption

**Figure 1:**
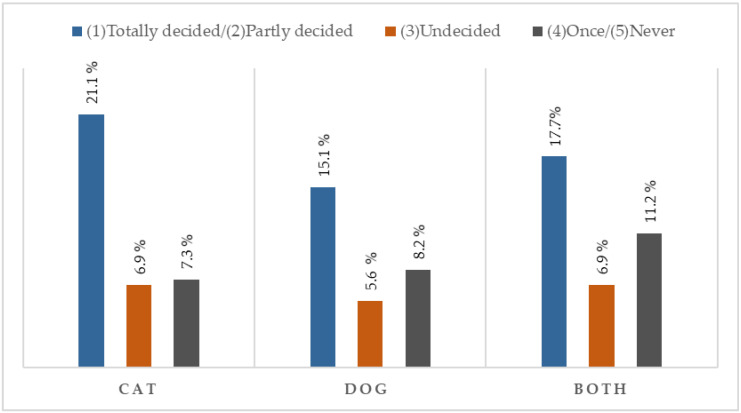
Q15.Would you feed your pet cultured meat-based food?

Figure 1 illustrates the differences in attitudes between cat and dog owners regarding their openness to cultivated meat-based food, based on responses to Question Q15: Would you feed your pet cultured meat-based food?

## 4. Discussions

Regarding the socio-demographic profile of respondents, the gender influence is particularly relevant. We are aware that in online studies, the female population is often better represented numerically, an aspect that is also highlighted in our research. This phenomenon is also confirmed by the literature, which shows that women participate more frequently in online surveys [25]. This increased representation of women is not just a methodological effect but also reflects the broader social and economic reality. Internationally, women play a central role in making decisions related to food for both people and pets [26] in a household, as shown by several socio-demographic and gender studies [27,28].

Our results suggest that women are more involved in food-related decision-making than men, which is consistent with trends observed in European studies. However, these findings should be interpreted with caution due to the overrepresentation of women in the sample.

The comparative analysis between women and men regarding the perception of cultured meat (Q8) reveals a statistically significant difference between the two groups (*p* = 0.003), according to the ANOVA test. The scale used is an ordinal one, ranging from positive perceptions (1–3) to negative or uncertain perceptions (4–6), as shown in Table 2, so that lower values reflect a more favorable attitude. The average score for women is 2.368, indicating a tendency towards positive or moderate perceptions, particularly towards options such as “a solution for food security” or “an innovation beneficial to the environment”.

In contrast, the higher average score among men (3.086) indicates a slightly more neutral or even critical perception—for example, “ethical alternative” or initial doubts about the naturalness of the product. Also, positive Skewness among women (0.756) indicates a slight asymmetry towards more favorable perceptions, while men have an almost symmetrical distribution (Skewness ≈ 0.070), which signals greater diversity in responses.

The results show that within our sample, women expressed somewhat more favorable and homogeneous perception of cultured meat, while men showed greater variability, with a slightly higher average, suggesting a more neutral or ambivalent position. Although this difference reached statistical significance, it should be interpreted with caution and seen as exploratory, offering only an indicative perspective on the potential influence of gender in the acceptance of new food technologies. In review studies, other authors note, contrary to the results of the current study, that men tend to be more tolerant of accepting cultured meat in comparison to women [18,29], but the difference is subtle and inconsistent across studies. Respondents’ willingness to try and eat was higher in men than in women. Dupont et al. [24] conducted a similar study in Germany and did not identify any differences in acceptance between genders.

In order to identify differences in motivation when choosing and purchasing cultured meat (question Q9), the analysis was conducted separately for women and men, and the response scale was ordered so that a value of (1) scale (Table 2) indicates confidence in scientific evidence regarding product safety, considered the most rational and objective reason, while higher values reflect more subjective motivations (taste, price, social recommendations).

The average score is significantly lower among men (1.655) compared to women (2.103), indicating that men are more strongly influenced by scientific arguments, while women tend to consider other important criteria, such as taste or environmental impact. This difference is supported by the statistical test result, with a *p*-value of 0.030, which confirms the existence of a statistically significant difference between the two groups.

The distribution of responses provides additional information about the behavior of respondents: the positive Skewness scale in both groups, more pronounced in men (1.511) than in women (0.648), indicates a tendency for responses to cluster around the value (1) scale (Table 2). The negative Kurtosis in women suggests a distribution leaning towards greater variance, while in men it is slightly more concentrated.

When it comes to motivations for purchasing cultured meat products, recent research shows that women more often cite concerns about animal welfare and environmental issues, while men are mainly motivated by curiosity or the technological process [29,30]. Our research confirms these findings, which have been studied in populations in other European countries.

With regard to the use of cultured meat in pet food (Q16), lower values on the scale indicate positive attitudes towards the ethical and ecological benefits of this option, while higher values reflect skepticism or disinterest. The results show a clear difference between the two groups. Women have an average response of 1.839, compared to 2.259 for men, suggesting a stronger appreciation of advantages such as no animal sacrifice and reduced environmental impact among female respondents.

The distribution of responses is slightly asymmetrical in both groups, more pronounced in women (Skewness = 1.378) than in men (0.806), reflecting a more pronounced tendency for women to choose options at the favorable end of the scale. The Kurtosis values indicate a relatively normal distribution in women (0.620), but flatter in men (−1.119).

The statistical significance of this difference is supported by the test result, with a *p*-value of 0.044, indicating a statistically significant difference between the two groups. Therefore, we can say that gender significantly influences the perception of the advantages of cultured meat in pet food, with women showing greater openness and appreciation of these benefits.

Nevertheless, no studies analyzing gender differences in the acceptability of cultured meat for pet food have been identified in the literature to date. The only relevant study, conducted by Oven et al. [26], analyzed the general perceptions of pet owners and notes that very few respondents cited ethics or environmental impact as their main motivation, indicating that these issues are not perceived as significant differentiators from conventional pet food.

Regarding environmental influence, international literature suggests that urban populations may be more receptive to cultured meat than rural populations, particularly due to greater concerns about environmental impact [31]. However, as in our study, product safety was a common concern for both groups.

In our sample, which predominantly included respondents from urban areas, perceptions, knowledge, and attitudes towards cultured meat are relatively similar between the two populations, with one notable exception: in question Q16, which analyzes the advantages of cultured meat-based food for pets, where a statistically significant difference was identified (*p* = 0.004). Respondents in urban areas showed a significantly greater inclination towards the ethical aspects of food (no animal slaughter), while those in rural areas chose more varied answers, such as environmental impact or nutritional value. Given the composition of the sample, these findings should be interpreted with caution and seen as exploratory.

Oven et al. [26] conducted a comprehensive analysis of animal owners’ perceptions of cultured meat, one of the few studies to compare perceptions of cultured meat for human consumption and for pets. The study included demographic factors such as gender, age, and diet type, but no differentiation was made between rural and urban respondents.

In the case of age influence, based on Table 5, an exception appears in question Q9, where there is a significant difference between the 18–24 and 35–44 age groups (*p* = 0.029), suggesting a variation in motivation to try cultured meat depending on age. This specific finding may indicate that young people are more open to food innovations, an aspect also highlighted in other international studies [32,33], but it should be interpreted with caution, given the exploratory nature of the research and the lack of other consistent differences between groups.

In a similar empirical study with respondents in the US, researchers made observations about young people’s behaviour regarding their perception of cultured meat, noting that they act from a general attitude of openness and prosociality, while older people respond more strongly to emotional stimuli such as regret [32]. Basically, what has been demonstrated is that those in the younger age groups are more open to the idea of cultured meat from the outset but are more motivated by ethical reasoning and innovation, while older age groups are less willing to accept food innovations initially but respond more strongly to emotional stimuli such as regret for passivity [33] and the desire to do something valuable, which may prompt them to try cultured meat.

Regarding European countries, Bryant et al. [18] found that cultured meat was more widely accepted among young people in Germany and France. Similar results were observed in a study conducted in Poland [34], where the authors identified the same trends. While these findings are partially consistent with our observations from question Q9, more conclusive results require studies of populations with more balanced age distributions and larger sample sizes.

In the section perception of cultured meat (human consumption), based on Table 2, the results indicate a high level of familiarity among respondents with the concept of cultured meat as an alternative source of protein. When asked, ‘Have you heard of cultured meat?’, 70% of respondents answered affirmatively, suggesting significant exposure to this topic even before it was commonly introduced on the Romanian market. Nonetheless, these results cannot be generalized at the national level, as the sample is not representative of Romania’s entire population.

A recent study conducted by YouGov and GFI Europe [10] analyzed the level of familiarity with cultured meat among the population of 15 European countries. Participants were asked if they had heard of this innovative method of meat production, and the results showed significant differences between countries. The highest levels of familiarity were recorded in the Netherlands, Austria, Sweden and Germany, while in Romania only 25% of respondents said they were familiar with this concept. This discrepancy could be explained by the socio-demographic characteristics of the sample analyzed in the current study, which was composed of over 60% of people with higher education (bachelor’s degree or higher), as it is well known that the level of education influences the degree of knowledge and openness to food innovations.

It should also be noted that the current study was mainly conducted in the north-eastern part of Romania, and the sample does not uniformly reflect the structure of the entire national population, which may contribute to the differences observed in terms of familiarity with this topic. Consequently, the results should be interpreted with caution and viewed as exploratory for this specific category of consumers.

Regarding the question ‘What would convince you to buy cultured meat?’, the answers were analyzed according to the order in which the options were voted by the respondents (using a scale numbered from 1 to 6), as can be seen in Table 2. The most convincing condition was the existence of scientific evidence regarding the safety of the product, selected by 40.6% of respondents as their first choice. In second place was a taste similar to that of conventional meat, selected by 24.4% of respondents, followed by environmental benefits. Other motivations, such as affordable price and recommendations from experts or friends, were significantly less influential. These results highlight that perceived safety and familiarity of taste are essential factors for the potential acceptance of cultured meat among consumers in Romania.

A previous study conducted in Romania on consumer attitudes towards alternative protein sources provided relevant data on the population’s openness to such products [35]. In this study, a sample of 178 respondents was surveyed about the likelihood of adopting alternative protein sources in their diet. The results showed a low level of acceptance, with only 6.8% of participants stating that they were very likely to make this transition. In contrast, a significant cumulative proportion, approximately 69.1%, indicated that it was unlikely or very unlikely that they would include such products in their diet. These data suggest a general reluctance among Romanian consumers towards alternative proteins, which can be explained by factors such as lack of information, attachment to traditional foods, or perceptions about the safety and taste of new protein sources, as highlighted in the current study.

In the section perception of cultured meat for animal consumption, based on Table 2, the first question was designed to segment respondents according to whether they owned a pet, in order to ensure the relevance of the subsequent questions. Of the total 233 respondents, 71 said they owned a dog, 78 a cat, and 84 respondents owned both. This initial filtering allows for a differentiated investigation of the degree of acceptance of cultured meat, both in terms of personal consumption and animal consumption—or even the simultaneous acceptance of both forms of use.

Based on the data obtained in our research on the level of awareness and information of pet owners regarding the food they feed to their pets, the results indicate that 50.5% of respondents say they always read the label on pet food products, while only 3.7% say they never read it. This behavior could reflect either a high educational profile of the sample or increased emotional involvement, specific to people who consider pets as members of the family.

In comparison, international studies report higher percentages: between 90% and 92% of owners say they check the list of ingredients when purchasing pet food [36]. However, specialist research draws attention to a discrepancy between declared behavior and the actual level of understanding of the information on the label [37,38].

Given the international context, in England, over 50% of dog owners and 62% of cat owners expressed a positive attitude towards adding insects to their pets’ diets after being informed about the nutritional and sustainability benefits [39]. However, with regard to vegetarian diets for pets, Dodd et al. [40] show that although just over half of pet owners expressed concerns about conventional meat-based food, almost all reported significant concerns about plant-based food. These concerns relate in particular to nutritional safety and the potential impact on animal health, suggesting major reluctance to adopt vegan or vegetarian diets in the absence of solid scientific evidence.

In this study, 91% of respondents said they feed their pets conventional meat-based products (chicken, beef, pork, etc.), and a very small percentage (3.4%) use insect-based food. Although these percentages were not the main focus of the research, they do provide an indication of consumers’ openness to alternative sources of protein. This attitude may also be relevant to understanding receptivity to cultivated meat, which falls into the same category of sustainable food innovations. Market research data confirms the choices expressed in our study, with most respondents opting for well-known flavors and protein sources commonly found in pet food brands. Hence, between July 2023 and June 2024, the most popular flavours purchased were chicken and beef (16.4%), beef (15.8%), and mixed meat (12.1%), and the largest increase in market share in terms of value was recorded by the beef with vegetables flavor, with a 48.9% increase compared to the previous period (July 2022–June 2023) [41].

Two important findings of this study highlight the relatively high degree of openness of respondents to the use of cultured meat in pet food. Thus, 33.2% of participants said they were “totally decided” to feed their animals cultured meat products, and another 21% chose the “partially decided” option, outlining a positive trend. In addition, over 70% of respondents said they would agree with the introduction of cultured meat on the market, both for human consumption and for animals, provided it is approved by the competent legislative authorities. These findings, though, reflect the characteristics of a specific sample, mostly young, urban, and highly educated, and cannot be generalized to the entire Romanian population.

Compared to the low acceptance of alternative protein sources, such as insect-based pet food (chosen by only 3.4% of respondents) or 6.8% for human consumption in another study conducted in Romania, these results suggest that a food alternative that preserves the organoleptic and nutritional characteristics of conventional meat is much more easily accepted by the public in northeastern Romania. Similar studies are reported internationally, where acceptance of insect-based pet food remains limited due to neophobia and concerns about the naturalness of these diets, despite growing interest from research and the marketplace [42,43]. A more comprehensive analysis of alternative proteins in pet food also indicates that insect proteins face stronger barriers compared to cultured meat options [44].

Regarding Figure 1 (Question Q15), in the sample studied, cat owners showed the highest level of acceptance (totally or partially decided), while dog owners were more cautious, a trend that aligns with observations in the literature, according to which dog owners pay greater attention to the quality and safety of their pets’ nutrition [45]. Owners who have both cats and dogs more frequently opted for the answers “once” or “never,” possibly for the same reason: extra caution in feeding dogs. These results suggest that perceptions of innovative pet food may be influenced by the type of pet, but further studies with more representative samples are needed to confirm this trend.

### The Economic Context of the Romanian Pet Food Market

According to data provided by Market Data Forecast [46], which is based on market analyses integrated with sources such as compound annual growth rate (CAGR), FEDIAF (The European Pet Food Industry Federation) [47] and The European Consumer Organisation [46,48], the average price of premium pet food in Europe is estimated at approximately €10/kg. This price level is considered high in the economic context of Eastern European countries, according to the same sources.

As for the Romanian market, the online platform Zooplus [49] is identified as the main distributor of dog and cat food, with a significant share in the e-commerce sector. In our own analysis of the existing offer on this platform, which included representative dry dog and cat food products from the most popular brands in Romania, we found that the price per kilogram varies between €2 and €12. Some premium food manufacturers offer, according to the information they provide, products available at a minimum price of €2/kg, despite their commercial positioning in the premium segment.

This price variation highlights both the degree of economic accessibility and the diversity of the market, which are essential aspects in analyzing consumer behavior and assessing the potential for acceptance of innovative alternatives, such as cultured meat, in pet food in Romania.

According to the data obtained, the majority of respondents (50.5%) would be willing to pay less than RON 50/kg or €10 for cultured meat-based pet food, thus aligning with current prices for conventional pet food. However, a significant percentage, approximately one-third of respondents, would be willing to pay a higher price, suggesting an understanding that a new, sustainable, and ethical source of protein may come at a higher cost.

Evaluating these data, as well as the willingness expressed by some respondents to purchase pet food at higher prices, there is a clear trend towards higher quality or premium products. This trend is also supported by international market analysis, which shows that in 2024, pet food sales in Romania increased by almost 10% compared to the previous year [50]. However, Romania continues to record a significant trade deficit in this sector, ranking fourth worldwide with a negative balance of $363 million—surpassed only by the United Kingdom, Japan, and Canada, according to data from the World Top Exports platform [51].

The results of the current study indicate that the main challenge in accepting cultured meat is the lack of clear information, identified by 75.1% of respondents. This conclusion highlights the need for accessible, transparent information campaigns supported by official sources (legislative authorities, scientific institutions) to ensure that the public has a correct understanding of the production process, safety, and nutritional benefits of this type of product. The management of pet nutrition is strongly influenced by external factors such as owner attitudes, product accessibility, and socio-economic contexts, as has been highlighted in studies on weight control programs for dogs and cats [52]. In this regard, the introduction of cultured meat-based food could encounter similar influencing factors, suggesting that acceptance does not depend solely on product characteristics, but also on the external environment and consumer perceptions.

Furthermore, there is a correlation between this need for information and the answers given to question Q9, where scientific evidence regarding product safety and similarity in taste to conventional meat, also found in other similar studies [53], are the strongest motivational factors for acceptance [54]. Therefore, the lack of clarity of information becomes not only a barrier to perception, but a direct obstacle to the purchase decision.

The lack of clear information on the process of producing cultured meat, its nutritional adequacy, and food safety are major barriers perceived both by the public in our sample (characterized by easy access to online information and a high proportion of young respondents) and, in addition, by the general public. In this context, we consider information and documentation initiatives to be useful, such as the use of simplified infographics explaining the technological stages of obtaining cultured meat or the organization of thematic meetings supported by legislative authorities in collaboration with professionals in the field of human and animal nutrition and feeding. Such actions could help build consumer confidence and, at the same time, create opportunities for small businesses interested in entering the local market.

## 5. Conclusions

This exploratory study suggests a relatively favorable perception of cultivated meat among respondents, with 33.2% stating that they would definitely consider feeding it to their pets and over 70% supporting its introduction to the market, provided that it is legally regulated. Nevertheless, these findings reflect the characteristics of a specific sample, predominantly young, urban, and highly educated, and cannot be generalized to the entire population of Romania. A gender analysis revealed statistically significant results. Women displayed slightly more positive attitudes, emphasising ethical and environmental aspects, while men placed greater emphasis on scientific validation and product safety. While these patterns are limited to our sample, they may suggest that communication strategies for promoting cultivated meat could benefit from gender-specific adaptations—an ethical framework for women and a scientific one for men.

In terms of place of residence, no consistent differences were observed between urban and rural respondents, except for one item related to pet food, where urban respondents expressed stronger ethical motivations. Although exploratory, these results may suggest directions for adapting awareness campaigns. Future research in Romania could aim to expand the sample size to obtain a more representative picture at the national level regarding the acceptability of cultured meat. A promising direction would be to separately analyze the perception of human consumption versus animal consumption, in order to highlight differences in attitude depending on the purpose of consumption. In addition, a systematic comparison between cultured meat and other alternative sources of protein, such as protein from insects [55], algae, or plant products, is needed so that we can determine the degree of openness of the public to each category and identify which arguments, for example, nutritional, ethical, or ecological, would most influence consumer behavior.

## Figures and Tables

**Table 1:** Structure of the questionnaire: sections, questions, and available response options regarding cultured meat perception for human and pet consumption.

Section	Question	Answer
General, socio-demographic information	Age	18–24 years/25–34 years/35–44 years/45–54 years/55+ years
	Gender	Male/Female/Other/Prefer not to answer
	Environment	Urban/Rural
	Level of education	High school education Post-secondary education University education (bachelor’s degree) University education (master’s degree) PhD education
	Do you have a special diet?	No, I eat all types of meat. I am an ovo-lacto vegetarian. I am vegan, so I don’t consume any animal products. I am a flexitarian (I occasionally eat meat).
	Who decides what is consumed in the household:	Me/My partner/Someone else (female)/Someone else (male)
Perception of cultured meat (human consumption)	Have you heard of cultured meat?	Yes/No
	How do you perceive cultured meat?	An innovation that is beneficial to the environment An ethical alternative to conventional meat An unhealthy artificial product A solution that can help food security I am not interested/I would not consume it I am not informed.
	What would convince you to buy and try cultured meat?	Scientific evidence regarding its safety. Similar in taste to conventional meat. Environmental benefits Affordable price Recommendations from experts or friends.
Perception of cultured meat (animal consumption)	Do you have a pet?	Dog/Cat/Both
	Who decides what the pet eats?	Me/My partner/Someone else (female)/Someone else (male)
	What type of food do you usually feed them?	Dry food (kibble) Wet food (canned/pouches) Home-cooked food A combination of the above
	How often do you read the ingredient labels on pet food?	Always/Sometimes/Rarely/Never
	The food you give your pet is mainly based on:	Meat (poultry, beef, pork, etc.) Vegetarian (only based on grains and/or legumes) Insect protein
	Would you feed your pet cultured meat-based food?	Never/Once/Undecided/Partly decided/Totally decided
	What advantages would you consider important for cultured meat-based animal feed?	No animal slaughter Less environmental impact Lower risk of bacterial contamination Optimised nutritional intake No advantages, not interested
	How much would you be willing to pay for cultured meat-based pet food?	Less than 50 RON/kg Less than 100 RON/kg Less than 150 RON/kg I would not purchase it
Conclusions and opinions	Would you agree with the introduction of cultured meat on the market (if it were approved for consumption by the legislative authorities)?	Yes, for both humans and animals. Yes, only for humans Yes, only for animals No
	What do you think is the main challenge in accepting cultured meat?	Lack of clear information High price Consumer mistrust Lack of regulations
	Would you like to add an opinion on this topic?	Open answer

**Table 2:** Respondent distribution by perceptions and attitudes towards cultured meat.

Number	Question	Results	
Q1	Age	18–24 y	47.2%
		25–34 y	32.6%
		35–44 y	10.7%
		45–54 y	6.4%
		55+ y	3.0%
Q2	Gender	Female	75.1%
		Male	24.9%
Q3	Environment	Urban	75.5%
		Rural	24.5%
Q4	Level of education	High school education	35.2%
		Post-secondary education	2.6%
		University education (bachelor’s degree)	28.8%
		University education (master’s degree)	26.6%
		PhD education	6.9%
Q5	Do you have a special diet?	No, I eat all types of meat.	86.7%
		I am a flexitarian (I occasionally eat meat).	9.0%
		I am an ovo-lacto vegetarian.	2.6%
		I am vegan, so I don’t consume any animal products.	1.7%
Q6	Who decides what is consumed in the household:	Female/Someone else (female)	79.0%
		Male/Someone else (male)	21.0%
Q7	Have you heard of cultured meat?	(1) Yes	70.0%
		(2) No	30.0%
Q8	How do you perceive cultured meat?	(1) An environmentally beneficial innovation.	40.6%
		(2) A solution that can help with food security	13.4%
		(3) An ethical alternative to conventional meat	17.4%
		(4) An unhealthy artificial product	10.3%
		(5) I’m not interested/I wouldn’t consume it	14.3%
		(6) I am not informed.	4.0%
Q9	What would convince you to buy and try cultured meat?	(1) Scientific evidence regarding its safety.	42.3%
		(2) Similar in taste to conventional meat.	24.4%
		(3) Environmental benefits	14.3%
		(4) Affordable price	13.6%
		(5) Recommendations from experts or friends.	5.4%
Q10	Do you have a pet?	Dog	30.5%
		Cat	33.9%
		Both	35.6%
Q11	Who decides what the pet eats?	Female/Someone else (female)	74.2%
		Male/Someone else (male)	25.8%
Q12	What type of food do you usually feed them?	Dry food (kibble)	25.7%
		Wet food (canned/pouches)	4.7%
		Dry and wet food	27.1%
		Home-cooked food	2.8%
		A combination of the above	39.7%
Q13	How often do you read the ingredient labels on pet food?	(1) Always	50.5%
		(2) Sometimes	34.4%
		(3) Rarely	11.5%
		(4) Never	3.7%
Q14	The food you give your pet is mainly based on:	(1) Meat (poultry, beef, pork, etc.)	91.0%
		(2) Vegetarian (only based on grains and/or legumes)	5.6%
		(3) Insect protein	3.4%
Q15	Would you feed your pet cultured meat-based food?	(1) Totally decided	33.2%
		(2) Partly decided	21.0%
		(3) Undecided	17.5%
		(4) Once	15.3%
		(5) Never	13.1%
Q16	What advantages would you consider important for cultured meat-based animal feed?	(1) No animal slaughter	32.0%
		(2) Less environmental impact	27.1%
		(3) Lower risk of bacterial contamination	20.0%
		(4) Optimised nutritional intake	15.3%
		(5) No advantages, not interested	5.6%
Q17	How much would you be willing to pay for cultured meat-based pet food?	(1) Less than 50 RON/kg or 10 €	50.9%
		(2) Less than 100 RON/kg or 20 €	29.8%
		(3) Less than 150 RON/kg or 30 €	6.4%
		(4) I would not purchase it	12.8%
Q18	Would you agree with the introduction of cultured meat on the market (if it were approved for consumption by the legislative authorities)?	(1) Yes, both for humans and animals.	70.4%
		(2) Yes, for humans only.	2.4%
		(3) Yes, for animals only.	11.7%
		(4) No.	15.5%
Q19	What do you think is the main challenge in accepting cultured meat?	(1) Lack of clear information	75.1%
		(2) Consumer mistrust	16.3%
		(3) High price	6.9%
		(4) Lack of regulations	1.7%
Q20	Would you like to add an opinion on this topic?	Open answer	

**Table 3:** Descriptive statistics on perceptions of cultured meat by gender.

Question	Female				Male				
	Mean	St. Dev.	Kurtosis	Skewness	Mean	St. Dev.	Kurtosis	Skewness	*p*-Value
Q7	1.270	0.034	−0.920	1.045	1.397	0.065	−1.877	0.434	0.070
Q8	2.368	1.540	−0.741	0.756	3.086	1.668	−1.377	0.070	0.003
Q9	2.103	1.398	−1.378	0.648	1.655	1.207	0.611	1.511	0.030
Q13	1.741	0.844	0.527	1.046	1.759	0.997	−0.086	1.061	0.898
Q14	1.069	0.315	25.023	4.934	1.172	0.500	7.832	2.946	0.066
Q15	2.511	1.346	−1.031	0.443	2.569	1.476	−1.227	0.386	0.066
Q16	1.839	1.266	0.620	1.378	2.259	1.639	−1.119	0.806	0.044
Q17	1.741	0.972	0.464	1.228	1.897	1.135	−0.564	0.956	0.314
Q18	1.638	1.107	0.441	1.454	1.828	1.230	−0.557	1.102	0.273
Q19	1.322	0.627	2.923	1.913	1.448	0.841	2.785	1.908	0.226

**Table 4:** Descriptive statistics on perceptions of cultivated meat by environment influence.

Question	Rural				Urban				
	Mean	St. Dev.	Kurtosis	Skewness	Mean	St. Dev.	Kurtosis	Skewness	*p*-Value
Q7	1.316	0.469	−1.387	0.814	1.297	0.458	−1.212	0.895	0.791
Q8	2.684	1.671	−1.261	0.476	2.503	1.579	−0.964	0.602	0.459
Q9	2.018	1.445	−0.988	0.888	1.983	1.341	−1.178	0.798	0.868
Q13	1.912	0.892	0.469	0.959	1.691	0.875	0.381	1.114	0.101
Q14	1.158	0.492	9.031	3.153	1.074	0.322	22.643	4.688	0.141
Q15	2.544	1.310	−1.013	0.370	2.520	1.401	−1.099	0.447	0.910
Q16	2.404	1.510	−0.867	0.788	1.794	1.301	0.552	1.418	0.004
Q17	1.702	1.510	0.716	1.378	1.806	1.301	0.030	1.099	0.503
Q18	1.702	1.164	0.013	1.318	1.806	1.135	0.190	1.372	0.901
Q19	1.368	0.672	3.853	1.968	1.349	0.694	3.410	2.020	0.850

**Table 5:** Summary of statistically significant differences between age groups (Tukey’s test).

Question	Significant Comparison	*p*-Value	Direction of Difference
Q9	18–24 vs. 35–44	0.029	Younger respondents (18–24) scored higher

## Data Availability

All data used in this study are included in the explanatory tables and Supplementary Materials of this article. No new data were created or analyzed in this study.

## Supplementary Materials

The following supporting information can be downloaded from https://www.mdpi.com/article/10.3390/ani15192867/s1: Table S1. Full results of Tukey’s multiple comparison tests across age groups.
