# Supplementary material for: Consumer Acceptance of Cultured Meat in Romania Highlighting Sustainable Perspectives for Both Human and Pet Consumption
Source: Animals (Basel). 2025 Sep 30;15(19):2867. doi: 10.3390/ani15192867 (PMC12523586; doi:10.3390/ani15192867)
Supplement: Supplementary file 1 [file animals-15-02867-s001.zip › animals-3882815-supplementary.pdf]

Supplementary Table S1: Full results of Tukey's multiple comparison tests across age groups

Tukey test	Q7-18 vs. Q7-25	Q7-18 vs. Q7-35	Q7-18 vs. Q7-45	Q7-18 vs. Q7-55	Q7-25 vs. Q7-35	Q7-25 vs. Q7-45	Q7-25 vs. Q7-55	Q7-35 vs. Q7-45	Q7-35 vs. Q7-55	Q7-45 vs. Q7-55
**Mean Diff.**	-0.163	0.104	-0.003	0.121	0.267	0.160	0.284	-0.107	0.017	0.124
**P Value**	0.120	0.842	>0.9999	0.960	0.085	0.725	0.511	0.952	>0.9999	0.976
**Summary**	ns	ns	ns	ns	ns	ns	ns	ns	ns	ns
**Tukey test**	**Q8-18 vs. Q8-25**	**Q8-18 vs. Q8-35**	**Q8-18 vs. Q8-45**	**Q8-18 vs. Q8-55**	**Q8-25 vs. Q8-35**	**Q8-25 vs. Q8-45**	**Q8-25 vs. Q8-55**	**Q8-35 vs. Q8-45**	**Q8-35 vs. Q8-55**	**Q8-45 vs. Q8-55**
**Mean Diff.**	-0.645	-0.738	-0.885	-0.675	-0.093	-0.240	-0.030	-0.147	0.063	0.210
**P Value**	0.052	0.217	0.250	0.806	0.999	0.983	>0.9999	0.999	>0.9999	0.998
**Summary**	ns	ns	ns	ns	ns	ns	ns	ns	ns	ns
**Tukey test**	**Q9-18 vs. Q9-25**	**Q9-18 vs. Q9-35**	**Q9-18 vs. Q9-45**	**Q9-18 vs. Q9-55**	**Q9-25 vs. Q9-35**	**Q9-25 vs. Q9-45**	**Q9-25 vs. Q9-55**	**Q9-35 vs. Q9-45**	**Q9-35 vs. Q9-55**	**Q9-45 vs. Q9-55**
**Mean Diff.**	0.442	0.869	0.642	1.309	0.427	0.200	0.867	-0.227	0.440	0.667
**P Value**	0.176	**0.029**	0.403	0.089	0.635	0.984	0.468	0.985	0.938	0.809
**Summary**	ns	*	ns	ns	ns	ns	ns	ns	ns	ns
**Tukey test**	**Q13-18 vs. Q13-25**	**Q13-18 vs. Q13-35**	**Q13-18 vs. Q13-45**	**Q13-18 vs. Q13-55**	**Q13-25 vs. Q13-35**	**Q13-25 vs. Q13-45**	**Q13-25 vs. Q13-55**	**Q13-35 vs. Q13-45**	**Q13-35 vs. Q13-55**	**Q13-45 vs. Q13-55**
**Mean Diff.**	0.018	0.351	-0.209	0.505	0.333	-0.227	0.488	-0.560	0.154	0.714
**P Value**	>0.9999	0.374	0.909	0.579	0.471	0.892	0.625	0.293	0.994	0.389
**Summary**	ns	ns	ns	ns	ns	ns	ns	ns	ns	ns
**Tukey test**	**Q14-18 vs. Q14-25**	**Q14-18 vs. Q14-35**	**Q14-18 vs. Q14-45**	**Q14-18 vs. Q14-55**	**Q14-25 vs. Q14-35**	**Q14-25 vs. Q14-45**	**Q14-25 vs. Q14-55**	**Q14-35 vs. Q14-45**	**Q14-35 vs. Q14-55**	**Q14-45 vs. Q14-55**
**Mean Diff.**	0.056	-0.011	-0.091	0.109	-0.067	-0.147	0.053	-0.080	0.120	0.200
**P Value**	0.856	>0.9999	0.902	0.944	0.938	0.634	0.996	0.965	0.944	0.767
**Summary**	ns	ns	ns	ns	ns	ns	ns	ns	ns	ns
**Tukey test**	**Q15-18 vs. Q15-25**	**Q15-18 vs. Q15-35**	**Q15-18 vs. Q15-45**	**Q15-18 vs. Q15-55**	**Q15-25 vs. Q15-35**	**Q15-25 vs. Q15-45**	**Q15-25 vs. Q15-55**	**Q15-35 vs. Q15-45**	**Q15-35 vs. Q15-55**	**Q15-45 vs. Q15-55**
**Mean Diff.**	-0.088	-0.315	-0.021	-0.555	-0.227	0.067	-0.467	0.293	-0.240	-0.533
**P Value**	0.993	0.843	>0.9999	0.842	0.954	1.000	0.913	0.967	0.994	0.917
**Summary**	ns	ns	ns	ns	ns	ns	ns	ns	ns	ns
**Tukey test**	**Q16-18 vs. Q16-25**	**Q16-18 vs. Q16-35**	**Q16-18 vs. Q16-45**	**Q16-18 vs. Q16-55**	**Q16-25 vs. Q16-35**	**Q16-25 vs. Q16-45**	**Q16-25 vs. Q16-55**	**Q16-35 vs. Q16-45**	**Q16-35 vs. Q16-55**	**Q16-45 vs. Q16-55**
**Mean Diff.**	-0.156	-0.716	-0.570	-0.522	-0.560	-0.413	-0.366	0.147	0.194	0.048
**P Value**	0.941	0.129	0.555	0.864	0.392	0.822	0.961	0.998	0.997	>0.9999
**Summary**	ns	ns	ns	ns	ns	ns	ns	ns	ns	ns
**Tukey test**	**Q17-18 vs. Q17-25**	**Q17-18 vs. Q17-35**	**Q17-18 vs. Q17-45**	**Q17-18 vs. Q17-55**	**Q17-25 vs. Q17-35**	**Q17-25 vs. Q17-45**	**Q17-25 vs. Q17-55**	**Q17-35 vs. Q17-45**	**Q17-35 vs. Q17-55**	**Q17-45 vs. Q17-55**
**Mean Diff.**	0.163	-0.424	-0.464	-0.692	-0.587	-0.627	-0.855	-0.040	-0.269	-0.229
**P Value**	0.810	0.310	0.441	0.386	0.083	0.174	0.194	>0.9999	0.970	0.987
**Summary**	ns	ns	ns	ns	ns	ns	ns	ns	ns	ns
**Tukey test**	**Q18-18 vs. Q18-25**	**Q18-18 vs. Q18-35**	**Q18-18 vs. Q18-45**	**Q18-18 vs. Q18-55**	**Q18-25 vs. Q18-35**	**Q18-25 vs. Q18-45**	**Q18-25 vs. Q18-55**	**Q18-35 vs. Q18-45**	**Q18-35 vs. Q18-55**	**Q18-45 vs. Q18-55**
**Mean Diff.**	-0.018	-0.458	-0.618	-0.275	-0.440	-0.600	-0.257	-0.160	0.183	0.343
**P Value**	>0.9999	0.361	0.277	0.971	0.447	0.335	0.979	0.993	0.996	0.964
**Summary**	ns	ns	ns	ns	ns	ns	ns	ns	ns	ns
**Tukey test**	**Q19-18 vs. Q19-25**	**Q19-18 vs. Q19-35**	**Q19-18 vs. Q19-45**	**Q19-18 vs. Q19-55**	**Q19-25 vs. Q19-35**	**Q19-25 vs. Q19-45**	**Q19-25 vs. Q19-55**	**Q19-35 vs. Q19-45**	**Q19-35 vs. Q19-55**	**Q19-45 vs. Q19-55**
**Mean Diff.**	0.039	0.013	0.173	-0.199	-0.027	0.133	-0.238	0.160	-0.211	-0.371
**P Value**	0.996	>0.9999	0.893	0.947	1.000	0.960	0.907	0.954	0.953	0.766
**Summary**	ns	ns	ns	ns	ns	ns	ns	ns	ns	ns
